# Preparation of Chitosan/β-Cyclodextrin Composite Membrane and Its Adsorption Mechanism for Proteins

**DOI:** 10.3390/molecules28083484

**Published:** 2023-04-14

**Authors:** Tong Liu, Junbo Li, Hongyu Lei, Xinyu Zhen, Yue Wang, Dongxia Gou, Jun Zhao

**Affiliations:** College of Food Science and Engineering, Changchun University, Changchun 130022, China

**Keywords:** chitosan, β-cyclodextrin-based polymer, protein, adsorption performance, adsorption mechanism

## Abstract

A significant portion of the protein in food waste will contaminate the water. The chitosan/modified β-cyclodextrin (CS/β-CDP) composite membranes were prepared for the adsorption of bovine serum albumin (BSA) in this work to solve the problem of poor adsorption protein performance and easy disintegration by a pure chitosan membrane. A thorough investigation was conducted into the effects of the preparation conditions (the mass ratio of CS and β-CDP, preparation temperature, and glutaraldehyde addition) and adsorption conditions (temperature and pH) on the created CS/β-CDP composite membrane. The physical and chemical properties of pure CS membrane and CS/β-CDP composite membrane were investigated. The results showed that CS/β-CDP composite membrane has better tensile strength, elongation at break, Young’s modulus, contact angle properties, and lower swelling degree. The physicochemical and morphological attributes of composite membranes before and after the adsorption of BSA were characterized by SEM, FT-IR, and XRD. The results showed that the CS/β-CDP composite membrane adsorbed BSA by both physical and chemical mechanisms, and the adsorption isotherm, kinetics, and thermodynamic experiments further confirmed its adsorption mechanism. As a result, the CS/β-CDP composite membrane of absorbing BSA was successfully fabricated, demonstrating the potential application prospect in environmental protection.

## 1. Introduction

The release of food wastewater poses a severe threat to the environment, given the fast-growing food processing and manufacturing sectors [1]. Because of its huge discharge and complex composition, it is one of the key remediation projects in pollution prevention and control in China. One-tenth of China’s total sewage discharge is made up of food wastewater, which has chemical oxygen demand (COD) discharge and biochemical oxygen demand (BOD) discharge that is 10–100 times higher than those of regular urban sewage [2,3]. Food wastewater is usually the wastewater generated during food production, processing, washing, and cleaning. The pH of wastewater has an important influence on industrial corrosion because the pH of wastewater determines the amount of acidic or alkaline components in it, which affects the degree of corrosion of metals. When food industry wastewater is acidic, the pH value is below 7.0, in which acidic substances, such as organic and inorganic acids, accelerate the rate of metal corrosion, which is because acids can promote oxidation and corrosion reactions on metal surfaces, thus leading to corrosion of metal surfaces. Meanwhile, food wastewater has a high protein content and eutrophic pathogenic microorganisms, which leads to the rapid consumption of dissolved oxygen in the water and the death of fish and aquatic organisms [4]. Anaerobic reactions of protein pollutants result in the release of hydrogen sulfide (H_2_S), methane, and other reducing chemicals. Therefore, it is of great significance to remove protein before discharging wastewater to reduce pollutant discharge and improve the comprehensive utilization of water resources. Many methods, including the Isoelectric solubilization/precipitation method [5], and the adsorption [6], precipitator [7], and ultrafiltration methods [8], have been conducted in the last decade to treat protein from food wastewater. The adsorption method is often used due to its efficiency, easy application, and simple design. This has piqued the interest of experts in decreasing hazardous contaminants [9]. The key to the adsorption process is to select the appropriate adsorbent, which should contain the following characteristics: high adsorption capacity, fast adsorption speed, and easy separation or recovery from the treatment [10,11,12].

Deacetylation of the chitin (poly(2-amino-2-deoxy-D-glucose)) found in crustacean shells leads to the creation of chitosan (CS, a linear copolymer of (1–4)-linked 2-acetamido-2-deoxy-d-glucopyranose and 2-amino-2-deoxy-d-glucopyranose) [13]. The two amino groups and one hydroxyl group in the CS molecule make it very active [14]. Protein binding sites are provided by the amino groups in CS, which are protonated and positively charged in acidic conditions [15]. Intramolecular and intermolecular hydrogen bonds formed by hydroxyl groups with glycoside bonds or oxygen atoms of glycoside groups make CS readily film-forming [16]. The six-membered ring structure of the CS molecule makes it have good mechanical properties [17]. However, hydroxyl and amino groups are also strongly hydrophilic groups, which would result in poor hydrophobicity of CS membranes by thus limiting their application in wastewater. β-cyclodextrin-based polymer (β-CDP) retains the characteristic cavity structure of β-cyclodextrin (β-CD), which allows it to form host–guest complexes with various molecules through intermolecular forces, such as van der Waals forces and the hydrophobic effect. Additionally, due to its compatibility with both host and guest molecules, β-CDP can also form inclusion complexes with macromolecules, such as proteins [18,19]. This unique property of β-CDP makes it a versatile material with potential applications in various fields. In addition, β-CDP has low toxicity and good mechanical strength and stability [20,21]. The manufacture of CS/CDP composite membranes by cross-linking CS and CDP can increase their mechanical strength and adsorption effectiveness, and because of their low toxicity, they will not result in secondary wastewater pollution.

## 2. Results and Discussion

### 2.1. Optimization of Optimum Preparation Conditions of the CS/β-CDP Composite Membrane

#### 2.1.1. Single-Factor Experiment

**Figure 1:**
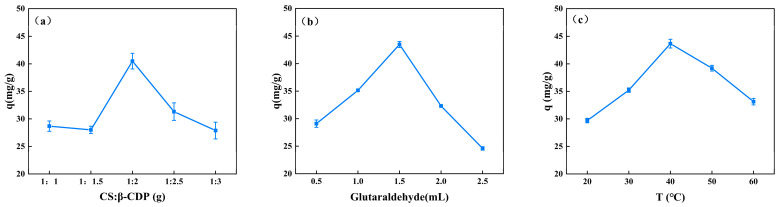
The effect of a single factor on the adsorption capacity of BSA. (**a**) CS: β-CDP, (**b**) glutaraldehyde addition, and (**c**) temperature.

The adsorption capacity of the composite membrane on BSA can be influenced by the mass ratio of CS to CDP, the quantity of glutaraldehyde, and the temperature at which the membrane is formed (Figure 1). The adsorption of BSA demonstrates a significant increase when the mass ratio of CS and β-CDP increases from 1:1 to 1:2, followed by a significant decrease at 1:2.5, as illustrated in Figure 1a. Notably, the mass ratio of 1:2 exhibits the highest adsorption capacity, which is significant at 40.49 ± 1.43 mg/g. The formation of inclusion complexes with BSA may account for the high adsorption capacity [19]. However, the effective sorption site combined with β-CDP is limited, and excessive β-CDP leads to a decreased membrane-forming property, thus reducing its adsorption capacity for BSA. The adsorption of BSA significantly increases as the glutaraldehyde addition increases from 0.5 mL to 1.5 mL and significantly decreases at 2.0 mL (Figure 1b). The glutaraldehyde addition in 1.5 mL shows a significantly higher adsorption capacity (43.48 ± 0.55 mg/g). It may be due to glutaraldehyde producing intermolecular hydrogen bonds that enhance the physical connection between the membrane components, resulting in a denser internal membrane structure [22]. However, excessive glutaraldehyde will occupy the -OH site of CS [23], reducing the effective adsorption site of membrane binding to BSA and decreasing the adsorption capacity of CS/β-CDP composite membranes of BSA. The adsorption of BSA significantly increases as the temperature increases from 20 °C to 40 °C and significantly decreases at 50 °C (Figure 1c). The temperature at 40 °C shows a significantly higher adsorption capacity (43.67 ± 0.79 mg/g). Therefore, within a certain temperature range, as the temperature increases, the rate of chemical reaction increases, which is similar to the previous report by Jing and colleagues [24]. The ability of CS/β-CDP composite membranes to absorb BSA will be reduced by high temperatures because they can break the chains of the CS molecules.

#### 2.1.2. Response Surface Model

To explore the adsorption capacity of BSA, BBD was chosen (Table 1), with three independent variables at three levels (high, low, and middle), and the data were used for model fitting (Table 2). The adsorption capacity of BSA (Y) and the coded values of the study components are shown by numbers from one to three based on the findings.

Y = 44.64 + 2.27A + 1.06B − 2.55C + 2.52AB − 3.37AC + 4.46BC − 20.79A^2^ − 9.70B^2^ − 13.63C^2^ (1–3)

The ANOVA results are summarized in Table 3. According to the models’ acquired F-value and *p*-value, the models are significant at a level less than 0.05%, the lack of fit is not significant at a level greater than 0.05%, and R^2^ is 0.9862. When the F-value is comparatively greater and the *p*-value is smaller in the variance analysis evaluation, then the more flexible the model is [25]. The results revealed that the model could be well used to explain the mass ratio, glutaraldehyde addition, and temperature as the BSA adsorption capacity in the conditions of the selected factors [26,27]. According to the *p*-value, A, C, D, AC, BC, A2, B2, and C2 variables are statistically significant (*p* < 0.05). The order of the effect of three factors on the adsorption capacity of BSA is C > A > B. The order of the effect of the mutual factors on the adsorption capacity of BSA is BC > AC > AB.

**Figure 2:**
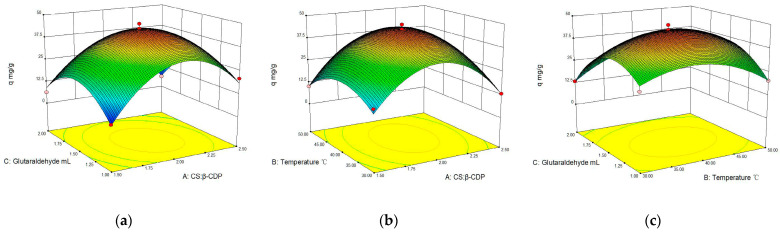
Effects of interaction of various factors on BSA adsorption: (**a**) glutaraldehyde addition and temperature; (**b**) glutaraldehyde addition and mass ratio of β-CDP and CS; and (**c**) mass of β-CDP and CS ratio and temperature.

The interaction between two parameters on BSA adsorption capacity with membranes was usefully explained by a 3D diagram using response surface methods (Figure 2). An arch is depicted in three dimensions from the lowest point to the highest point. BSA adsorption capacity increases with the mass ratio, temperature, and glutaraldehyde and then decreases in a parabolic manner [26]. The interaction between the two factors is comparatively more significant. The steeper the slope of the response surface, the more inclined, the more elliptical the contour, and so forth [28,29]. It is possible to create conditions that are economically optimal by analyzing the key elements and the influence of their interconnections. The optimum technological conditions were determined as follows: the mass ratio of CS and β-CDP of 1:2, a temperature of 40 °C, and glutaraldehyde of 1.5 mL. Under the optimum reaction condition, RSM predicted the BSA max maximum adsorption capacity is 44.2 ± 1.78 mg/g, and it was validated by experimental data generated (44.2 ± 1.56 mg/mL). The obtained results revealed an acceptable agreement between the experimental values and the predicted values; hence, the model can effectively predict the responses. Its results are higher than that of Feng [30] (protein adsorption capacity of 30 mg/g using chitosan-based amphiphilic membranes), indicating that the introduction of β-CDP was conducive to the adsorption of BSA.

### 2.2. SEM Analysis of the CS/β-CDP Composite Membrane before and after the Adsorption of BSA

**Figure 3:**
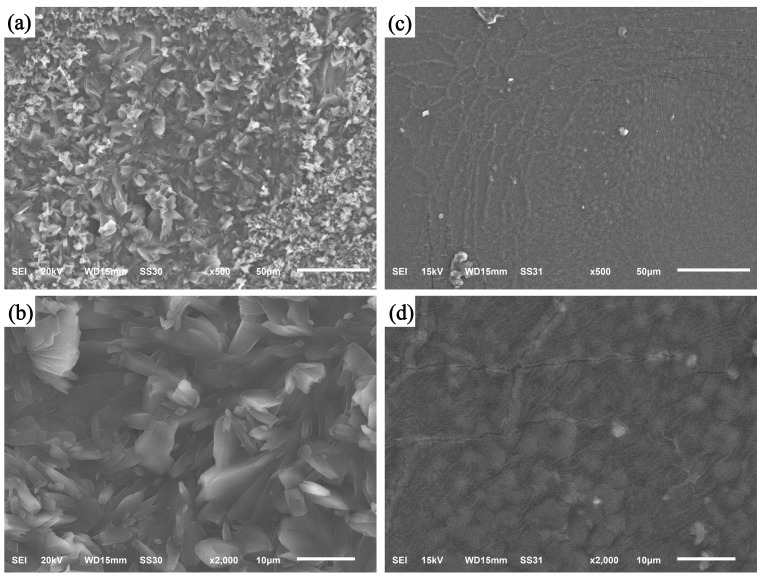
SEM images of CS membrane and CS/β-CDP composite membrane: (**a**) 500 times before adsorption of BSA; (**b**) 2000 times before BSA adsorption; (**c**) 500 times after BSA adsorption; and (**d**) 2000 times after membrane adsorption of BSA.

The SEM was utilized to examine the morphology of the CS/β-CDP composite membrane before and after BSA adsorption (Figure 3). The morphology of the prepared membranes is relatively rough (Figure 3a,b), with a large number of protrusions on them. This may be due to the exposure of hydroxyl and amino groups to the addition of glutaraldehyde, which induces their fusion with the protein. Although the distribution of pores in the CS/β-CDP composite membrane is not uniform, it still has a large specific surface area and adsorption space, which is similar to the previous report by Verma and colleagues [31]. The surface of the BSA-adsorbed CS/β-CDP composite membrane is smooth (Figure 3c,d). The sample had several cracks and visible pore structure, which shows that the cavity structure on the membrane’s surface is filled and that the BSA is physically adsorbing to the composite membrane. 

### 2.3. The Analysis of FT-IR and XRD

#### 2.3.1. FT-IR

**Figure 4:**
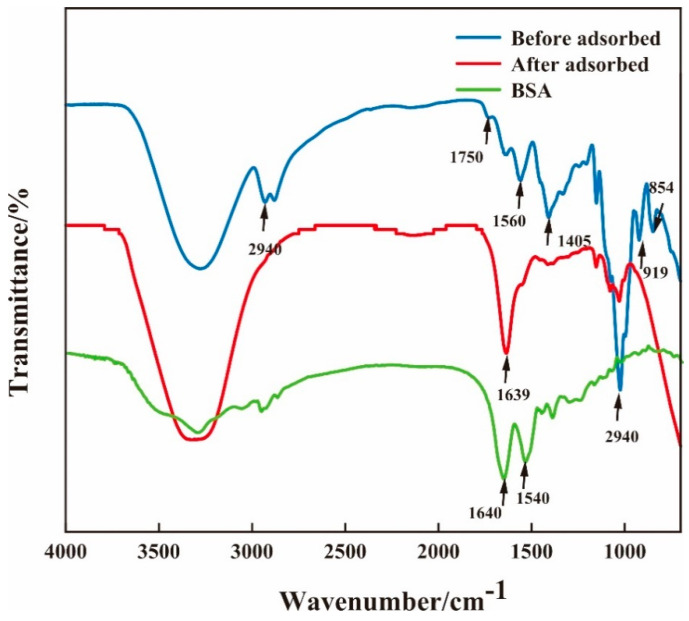
Changes of FT-IR before and after adsorption of BSA on the membrane.

Figure 4 shows the FT-IR spectra of BSA and the composite membrane before and after the adsorption of BSA. For the infrared spectrum of BSA, there is a strong absorption peak in the range of 1700–1600 cm^−1^ representing the carboxyl vibrations of cysteine, glutamic acid, and aspartic acid. In the range 1550–1480 cm^−1^, there is a strong absorption peak representing the aromatic ring C=C vibrations of tyrosine and phenylalanine. The above characteristic peaks are located roughly at 1640 cm^−1^ and 1540 cm^−1^ in the figure. The adsorption band around 854 cm^−1^ is a characteristic aspect of β-(1,4) glycoside bond of CS, the adsorption band around 919 cm^−1^ is a characteristic aspect of β-(1,4) glycoside bond of β-CDP [32], and the adsorption band around 1560 cm^−1^ is attributed to a large number of hydrogen bonds formed by glycerol and chitosan [33]. The adsorption band around 1725 cm^−1^ is attributed to Schiff base peak bonds formed by amino groups of chitosan and glutaraldehyde, which indicates that the cross-linking mode of CS and β-CDP are chemical cross-linking, which is similar to the previous report by Ulu and colleagues [34] for the membrane after BSA adsorption. Regarding the membrane after BSA adsorption, the absorption band observed at around 1022 cm^−1^ corresponds to the C–O stretching vibration of carbinol in the CS molecule [35]. The adsorption band around 1405 cm^−1^ is the C−N stretching vibration of methylene [36], and the weakening of peak intensity indicates that the structure of CS has changed. The 1560~1640 cm^−1^ is the mixed adsorption band, which is a characteristic peak of protein and mainly consists of amide I, amide II, and -NH2. It indicates that the BSA is adsorbed on the CS/β-CDP composite membrane. This result is similar to the findings of Sabaa and colleagues [37]. The peak of 2943 cm^−1^ is usually associated with a methyl (-CH3) functional group. The depletion of the 2943 cm^−1^ peak is observed on the CS/β-CDP composite membrane, which may imply that some chemical reactions occurred on the surface of the composite membrane. Meanwhile, the carboxyl group (-COOH) in BSA may be involved in this process, further suggesting that the adsorption process of BSA on the composite membrane is chemisorption.

#### 2.3.2. XRD

**Figure 5:**
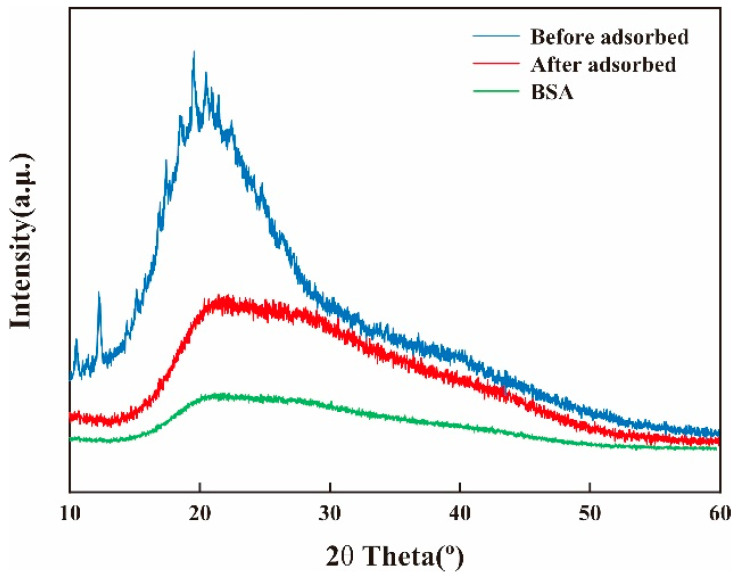
Changes of XRD before and after adsorption of BSA on the membrane.

Figure 5 shows the XRD of BSA with the membrane before and after the adsorption of BSA. The membrane had a diffraction peak around 2θ = 20°, which is mainly caused by the dissolution of CS in acetic acid solution [38]. CS/β-CDP composite membrane shows a sharp peak at 2θ = 12.5°, which is the crystal structure of β-CDP with a large number of oxygen-containing groups, which leads to hydration [39,40]. The relatively strong crystallinity and relatively high compactness of the membranes are attributed to the relatively regular structure of the membrane molecules, as well as the strong intermolecular and intramolecular hydrogen bonds, which was similar to the previous report by Sirajudheen and colleagues [41]. Combined with the diffraction pattern of BSA, it was found that the position of the diffraction peak did not significantly change after adsorption on the CS/β-CDP composite membrane, indicating that the crystal form did not change before and after adsorption of the BSA on the composite membrane, with uniform molecular chain and high crystallinity. After BSA is adsorbed, the crystallinity decreases because BSA forms hydrogen bonds with the membrane’s active group NH2, showing that more hydrogen bonds between the intramolecular and intermolecular networks are broken. 

### 2.4. Effects of Adsorption Temperature and pH on Adsorption Capacity of BSA

#### 2.4.1. Temperature

**Figure 6:**
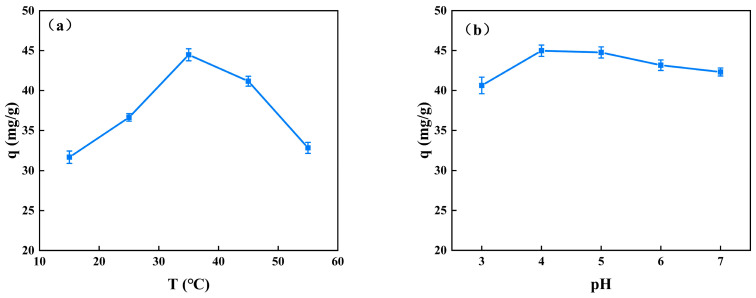
Effects of adsorption temperature and pH on the adsorption capacity of BSA: (**a**) adsorption temperature; (**b**) adsorption pH.

The uptake of BSA significantly increases from 15 °C to 35 °C when the solution pH is 4 and significantly decreases at 45 °C (Figure 6a). The temperature of 35 °C shows the significantly highest adsorption capacity (44.48 ± 0.75 mg/g). As long as a temperature rise promotes the BSA molecules’ Brownian motion, which is strengthened, the BSA molecules can easily connect with the efficient sorption sites of the CS/β-CDP composite membrane [42], which is similar to a previous report by Bazzaz and colleagues [43]. It can be explained as follows: The intense thermal molecular motion results in an increase in the internal disorder of BSA, thereby increasing the likelihood of intermolecular collisions. The CS/β-CDP composite membrane is susceptible to water loss, which can cause a structural collapse due to the reconfiguration of molecular chains.

#### 2.4.2. pH

When the temperature is constant at 37 °C, there is no significant effect on the adsorption of BSA when the pH is in the range of 3–7 (Figure 6b). The adsorption capacity of the carboxymethyl cellulose membrane prepared by Lin [44] for proteins decreased with increasing pH, which can be explained by the attraction of negatively charged proteins to carboxymethyl. CS is a cationic polymer with a certain degree of positive electrical properties. When forming a composite membrane with β-CDP, the positive electrical properties of the composite membrane are further enhanced by the interaction with the nucleophilic functional groups, such as amino and hydroxyl groups in CS, because the molecular structure of β-CDP contains electrophilic functional groups, such as a benzene ring and hydroxyl group. Therefore, the adsorption of BSA may be influenced by charge attraction and repulsion. Specifically, when the pH of the solution is low, the negative charge on the surface of BSA increases, leading to a stronger interaction with the positively charged CS/β-CDP, thus increasing the amount of BSA adsorbed in the solution. However, the molecular weight of BSA is relatively large, and its molecular structure is complex. Therefore, the adsorbed BSA molecules in the solution will not only interact with the surrounding ions but also be affected by other factors, such as hydration and ionic solution strength. These factors affect the conformation and spatial arrangement of BSA molecules in solution and thus affect the adsorption of BSA in solution. Experiments have shown that although changes in the pH of CS/β-CDP solutions can affect the amount of BSA adsorbed in the solution, this effect is usually small, especially in a certain range. This is because, within a certain range, factors such as ionic strength and hydration in the solution can have a balancing effect on the adsorption of BSA, making the change in solution pH less effective on the adsorption of BSA. Therefore, in industrial production, good protein adsorption can be achieved without modifying the pH of the solution.

### 2.5. The Analysis of Mechanical Properties

As shown in Table 4, the tensile strength of pure CS membrane and CS/β-CDP composite membrane is 4.95 ± 0.22 MPa and 6.37 ± 0.31 MPa, the elongation at break is 2.68 ± 0.17% and 3.79 ± 0.56%, and Young’s modulus is 5.12 ± 0.67 MPa and 7.56 ± 0.78 MPa, respectively. These three characteristics of the CS/β-CDP composite membranes are better than those of pure CS membranes, which suggests that glutaraldehyde has a strengthening impact on the CS/β-CDP and CS polymer matrix, in line with a recent report by Nie and colleagues [36]. This is mostly due to the electrostatic interaction of glutaraldehyde between CS and β-CDP, which enhances polymer chains’ flexibility, swelling, and susceptibility to fracture, which reduces mechanical resistance [45,46]. The higher tensile strength indicates that CS/β-CDP composite membrane was less prone to fracture than the CS membrane, and the elongation at break indicates that CS/β-CDP composite membrane has better plasticity [47], and Young’s modulus indicates that CS/β-CDP composite membrane has better deformation [48]. The results shed further light on the possibility of tuning the physicochemical properties of chitosan membranes by introducing β-CDP.

### 2.6. The Analysis of Contact Angle and Swelling Degree

The contact angles of water droplets and glycerol and the swelling in water were measured to study the wettability of CS/β-CDP composite and CS membranes.The contact angle can be calculated based on the membrane’s relative hydrophobicity [49]. Pure CS membrane has a contact angle of 66.43 ± 1.13°, showing a hydrophilic state. CS/β-CDP composite membrane has a contact angle of 91.23 ± 0.89°, showing a hydrophobic state. The same contact angle measurements with glycerol yield similar results to the water contact angle results, as shown in Table 5. This might be due to the enhanced interaction between CS and β-CDP, which lead to the formation of more hydrogen bonds and hence a decrease in the amount of -OH exposed on the surface of the membrane. This was consistent with the results of the swelling degree, which shows that the introduction of β-CDP can yield a membrane with good hydrophobicity, demonstrating its potential application prospect in the field of environmental protection.

The swelling degree can reflect the water adsorption capacity and pore structure of the membrane. Chitosan forms hydrogen bonds with water thanks to its hydroxyl and amino groups. Chitosan can accept water molecules by creating additional space due to its long chain structure [24]. The swelling degree for the CS membrane and CS/β-CDP composite membrane in water is found to be 71.06 ± 1.34% and 35.48 ± 1.27%, respectively. The result showed that the introduction of β-CDP significantly decreases the swelling degree. Compared with the surface of the CS membrane, the CS/β-CDP composite membrane exposes fewer hydrophilic groups. This might be due to glutaraldehyde occupying the adsorption sites of hydrophilic groups [50], which decreases its water adsorption capacity. Low swelling levels make it easier for the membrane to heal and be used. The differences in the surface free energy of the films calculated with the contact altimeter are also significantly small, with a maximum value of 32.02 mN/m when β-CDP is added at a concentration of 4%.

### 2.7. The Analysis of Kinetic Studies and Thermodynamics

#### 2.7.1. Adsorption Isotherms

**Figure 7:**
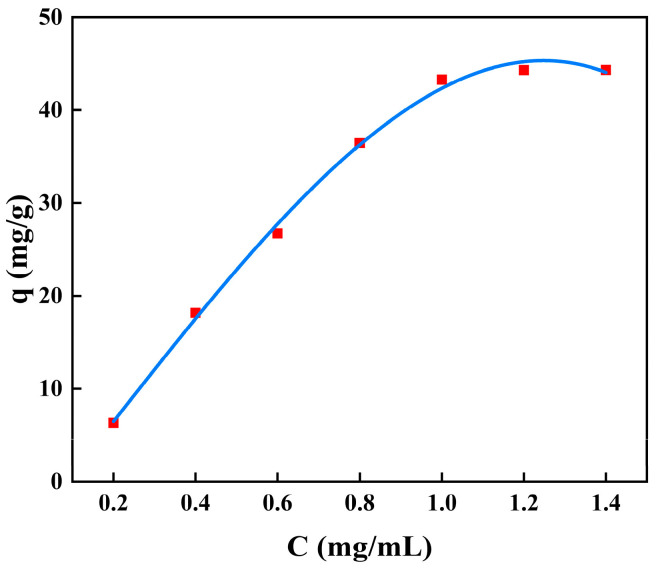
The model plot of nonlinear-fitted isotherm adsorption.

**Figure 8:**
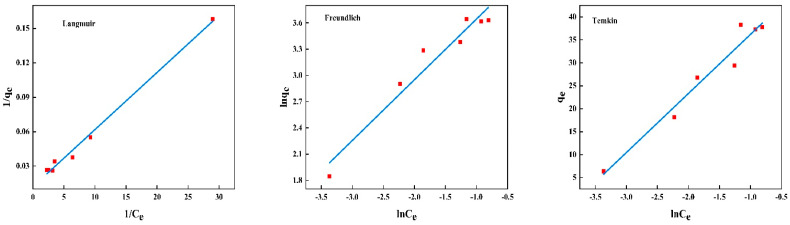
Model plot for linearly fitting isothermal adsorption.

The adsorption isotherms for BSA removal using CS/β-CDP composite membrane and the adsorption key parameters are tabulated in Table 6. The nonlinear fitting of BSA on the composite membrane is shown in Figure 7, and the linear fitting is shown in Figure 8. The R^2^ values of the Langmuir isotherm model, Freundlich isotherm model, and Temkin isotherm model are 0.9932, 0.9453, and 0.9565, respectively. BSA adsorption increases with the increase of initial BSA concentration and reaches equilibrium at 1 mg/mL. Based on the R^2^ value, the Langmuir isotherm was selected to describe the adsorption of BSA on the CS/β-CDP composite membrane. This result may confirm the monolayer adsorption of BSA onto the CS/β-CDP composite membrane surface [51,52]. The adsorption sites are evenly distributed on the surface [53,54], which is in direct contact with CS/β-CDP composite membrane [55]. From the Freundlich isotherm, K and 1/n indicate adsorption capacity at unit concentration and adsorption intensity, respectively. The K value is obtained at about 76.5237, and the n value is obtained at about 1.4407, indicating that CS/β-CDP composite membrane had a strong adsorption capacity for BSA [56,57], and it is spontaneous [25], which is similar to the previous report by Feng and colleagues [30]. When 0 < 1/n < 1, it indicates that the adsorption sites and strengths are non-uniformly distributed on the surface of the composite membrane [58]. The CS/β-CDP composite membrane has been found to exhibit multilayer adsorption of BSA on its outer surface [59,60]. The Temkin isotherm model assumes a linear relationship between the adsorption heat and the adsorption uptake of BSA [25]. The results of the Temkin model exhibit that there is a physical adsorption mechanism for BSA on the CS/β-CDP composite membrane, which is consistent with SEM results. Based on the R^2^ value, the order of the adsorption isothermal models of BSA on the CS/β-CDP composite membrane is as follows: Langmuir > Temkin > Freundlich.

#### 2.7.2. Adsorption Kinetics

**Figure 9:**
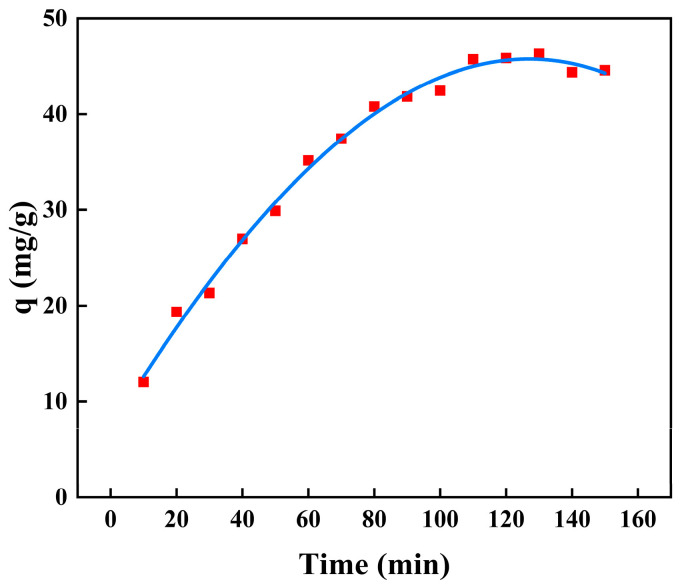
Nonlinear kinetics of BSA on the composite membrane.

**Figure 10:**
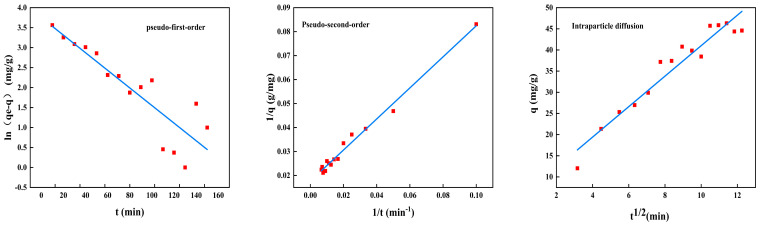
Kinetic adsorption model diagram.

The adsorption kinetics for BSA removal using CS/β-CDP composite membrane and the adsorption key parameters are tabulated in Table 7. The nonlinear fitting of the kinetic adsorption of BSA on the composite membrane is shown in Figure 9, and the linear fitting is shown in Figure 10. The R^2^ values of pseudo-first-order, pseudo-second-order, and intraparticle diffusion are 0.7806, 0.9874, and 0.9301, respectively. The adsorption data follows the pseudo-second-order kinetic model with R^2^ values close to 1.0 for BSA adsorption, which is evident that the chemisorption has occurred on CS/β-CDP composite membrane [55], which showed good agreement with FT-IR and XRD. BSA diffused from that surface to the internal adsorption site of the CS/β-CDP composite membrane and attaches to the membrane by covalent bonding with the active group on the membrane [49]. It can be seen from the intraparticle diffusion figure that the CS/β-CDP composite membrane had a high adsorption rate at the initial stage, which is a surface diffusion process. With the increase of time, the adsorption rate decreases, and intragranular diffusion occurs. However, the equation does not pass through the origin, indicates that intraparticle diffusion is not the only step for controlling the rate, indicating that BSA is not easily diffused inside that membrane [61], which is similar to the previous report by Huang and colleagues [62]. BSA is membrane-adsorbed on the CS/β-CDP composite membrane due to BSA and the functional groups on the surface of the CS/β-CDP composite membrane are combined through covalent bonding, an electrostatic interaction, van der Waals forces, a hydrogen bond, and other interactions. The adsorption rate of BSA is affected by chemical factors [63], such as the number and structure of functional groups on the membrane surface, BSA concentration, temperature, and the degree of the chemical reaction between BSA and the membrane [64]. Therefore, BSA adsorption kinetic could be controlled by a combination of intragranular diffusion and surface diffusion. Based on the R^2^ value, the order of the sequence of kinetics models of BSA on the CS/β-CDP composite membrane is as follows: pseudo-second-order > intraparticle diffusion > pseudo-first-order. 

#### 2.7.3. Thermodynamic

Adsorption thermodynamics for BSA removal using CS/β-CDP composite membrane and the adsorption key parameters are tabulated in Table 8. The negative value of ΔG demonstrates the feasibility and spontaneous nature of the process [65]. ΔG gradually decreases with increasing temperature, indicating that the spontaneous degree of the reaction also increases [66]. The positive value of ΔH indicates that BSA adsorption on CS/β-CDP composite membrane has an endothermic nature [67], and it is favored by temperature [68], which is similar to the previous report by Robert and colleagues [69]. Generally, if the heat of adsorption varies between 0 kJ/mol and 20 kJ/mol, the process is physisorption, whereas higher energy (80~200 kJ/mol) represents the chemisorption [70,71]. Intermediate values are related to systems wherein both interactions take place. The adsorption enthalpy of the CS/β-CDP composite membrane for BSA was 66 kJ/mol, between 20 kJ/mol and 80 kJ/mol. Thus, the BSA adsorption process could be considered between physical adsorption and chemical adsorption and prefer chemical adsorption. The results implied good agreement with FT-IR and XRD. The positive value of ΔS suggests that the entropy of this adsorption system is increased [72], indicating increased randomness at the solid/solution interface with an affinity of the CS/β-CDP composite membrane for BSA. Moreover, it corresponds to an increase in the degree of freedom of the adsorbed species and reflects that the distribution of BSA adsorbed on the membrane was more chaotic than that in the aqueous solution, which could be due to a combination of adsorbent with BSA [43].

## 3. Materials and Methods

### 3.1. Reagents and Instruments

Reagents: chitosan (biological reagents are usually >99% pure (BR)) was obtained from Sinopharm; β-cyclodextrin (BR) and absolute ethyl alcohol were obtained from Shanghai Huishi Biochemical Reagent, Ltd.; glutaraldehyde (analytical purity reagents are usually >98% pure (AR)) and glycerol were obtained from Tianjin Damao Chemical Reagent Factory; Cos Brilliant Blue G250 was obtained from Beijing Dingguo Changsheng Biotechnology, Lt.; citric acid (AR) was obtained from Macklin; and polyethylene Glycol 400 (AR), sodium dehydration phosphate (AR), glacial acetic acid (AR), and 85% phosphoric acid (AR) were obtained from Beijing Chemical Works.

Instruments: a TD-3000 X-ray diffractometer (Dandong Tongda Technology, Co., Ltd., Dandong, China); 3K15 centrifuge (Shanghai, China, Xima Centrifuge Ltd.); NTS-4000 Constant Temperature Oscillating Water Tank (Tokyo Physical and Chemical EYELA); Nicolis5ft-IR Fourier infrared spectrometer (Waltham, MA, USA, Thermo Fisher Scientific Shier Technology); IMark microplate reader (Hercules, CA, USA, bio-rad); SL200KS optical contact angle meter (Shanghai, China, Kono Industrial Co., Ltd.); and QJ210-50N Universal Mechanical Testing Equipment were used.

### 3.2. Methods

#### 3.2.1. Preparation of β-CDP 

The citric acid (2 g), polyethylene glycol 400 (1 g), sodium phosphate monobasic (0.25 g), and β-CD (10 g) were added to 80 mL ultrapure water, dissolved to transparency, and then dried at 145 °C for 4.5 h. The powder was taken out for grinding, a small amount of water to dissolve it was added, and then alcohol precipitation with 95% ethanol was made. We took the precipitate for filtration and drying and ground the dried solid powder.

#### 3.2.2. Preparation of the CS/β-CDP Composite Membrane

**Figure 11:**
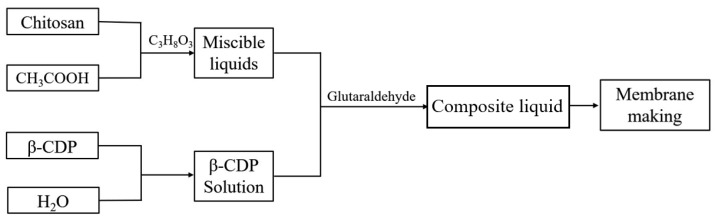
Flow chart of the preparation of CS/β-CDP composite membrane.

Figure 11 illustrates the process of preparing CS/β-CDP composite films using the flow-delay method. Chitosan (CS) at a concentration of 2% was dissolved in an acidic solution heated at 37 °C. At the same time, β-cyclodextrin-based polymer (β-CDP) at a concentration of 4% was dissolved in an aqueous solution. These two solutions were mixed in a 1:1 volume ratio. Next, glutaraldehyde was added to the mixture at a concentration of 0.25% by volume, and the resulting mixture was stirred well to ensure its homogeneity. Thirty g of the well-mixed solution was poured into a flat dish with a diameter of 9 cm and dried flat in a drying oven at 50 °C for 5 h to obtain the composite film.

#### 3.2.3. The Adsorption of BSA

The adsorption effect of the CS/β-CDP composite membrane of BSA was analyzed. A total of 1 g of CS/β-CDP composite membrane was placed in 10 mL 0.5 mg/mL BSA solution, and the solution was shaken at 100 r/min 1 h to determine the BSA content before and after shaking. The adsorption capacity of BSA was calculated using Equation (1). To ensure the reliability and accuracy of the experimental results and to reduce the uncertainty caused by experimental errors, three sets of parallel experiments were conducted for all our experiments.

(1) $$q=\frac{(C_{0}-C)V}{M}$$

where q is adsorption capacity (mg/g); *C*_0_ is BSA initial concentration (mg/mL); *C* is BSA supernatant concentration after adsorption experiment (mg/mL); *V* is BSA solution volume (mL); and *M* is composite film quantity (g).

#### 3.2.4. Single-Factor Experiment

Referring to the experimental method of Liu et al. [20], adsorbed BSA was carried out to examine the adsorption capacity of a CS/β-CDP composite membrane with different mass ratios (1:1, 1:1.5, 1:2, 1:2.5, and 1:3), temperatures (20, 30, 40, 50, and 60 °C), and glutaraldehyde additions (0.5, 1, 1.5, 2, 2.5, and 3 mL). The influencing factors and levels are shown in Table 9 below.

#### 3.2.5. Response Surface Methodology Experiment

The response surface optimization design: according to the results of the single-factor investigation, the BSA adsorption capacity was the response value, and the Box–Behnken design was used to conduct the 3-factor 3-level experiment.

#### 3.2.6. Effects of Adsorption Temperature and pH on the Adsorption Capacity of BSA

The optimum adsorption condition of BSA was determined by changing the temperature and pH. At the optimal membrane preparation conditions, the adsorption capacity of CS/β-CDP composite membrane for BSA was investigated at different temperatures (15, 25, 35, 45, and 55 °C) and pH (3, 4, 5, 6, and 7).

#### 3.2.7. Mechanical Performance Test

The mechanical properties of the membranes are controlled and tested by an electronic universal testing machine. The membranes were cut into strips measuring 10 mm × 70 mm and then tested with universal mechanical equipment at a stretching speed of 500 mm/min until the specimens broke. Ten specimens were tested under each condition, and the mean and standard deviation values of tensile strength, elongation at break, and Young’s modulus were calculated.

#### 3.2.8. Swelling Degree

The swelling was determined as described by Bagher [73] with some modifications. The membrane sample was precisely weighed (*m*_0_) and subsequently submerged in distilled water at ambient temperature for 24 h. The membrane was taken out of the water, its surface moisture was absorbed by filter paper, and the membrane was weighed (*m*_1_). The swelling degree of the membrane was calculated using Equation (2).

(2) $$\mathrm{Swelling}=\frac{m_{1}-m_{0}}{m_{0}}\times 100\%$$

where *m*_0_ is the initial mass of the sample film (g) and *m*_1_ is the mass of the sample membrane (g).

#### 3.2.9. Contact Angle Measurements

The water contact angle of the chitosan membrane surface was determined with optical contact angle meter. We added 5 μL droplets to the middle of the chitosan membrane and CS/β-CDP composite membrane. Then, the contact angle was measured after 30 s. Three parallel measurements were conducted, and the average value was determined. In addition to this, we measured the contact angle using glycerol and dimethylamine reagents and calculated the surface free energy (SFE) using Owens–Wendt.

#### 3.2.10. Scanning Electron Microscopy (SEM)

SEM analysis was performed to visualize the surface morphology prepared before and after adsorption of BSA by CS/β-CDP composite membrane using a scanning electron microscope. The membranes were cut into strips with a size of 0.3 mm × 0.3 mm, were gold plated for better conductivity, and then glued on an aluminum stub using double-adhesive carbon tape. The surface morphology was observed.

#### 3.2.11. FT-IR Analysis

FT-IR analysis was carried out to monitor the functional group of CS/β-CDP composite membrane before and after the adsorption of BSA. Samples and the controls were tested with a resolution of 2 cm^−1^ under the scan range of 600 cm^−1^ to 4000 cm^−1^.

#### 3.2.12. X-ray Diffraction (XRD) Analysis

XRD analysis was carried out to monitor the crystalline structure of the CS/β-CDP composite membrane before and after the adsorption of BSA. An angular step of 0.02° and a scanning speed of 2°/min were selected, and analyses were performed in a copper tube with a current voltage of 40 kV. The datum was collected in the range of 10–60° at 2θ. The crystallinity is calculated as described by Micheal [74] with some improvements using the Scherrer equation to calculate the crystallinity by measuring the half-height width of the peak in the XRD spectrum, as follows in Equation (3), where D is the average grain size of the crystal, K is a constant generally taken as 0.9, λ is the wavelength of the X-rays, β is the half-height width of the peak, and θ is the angle of the corresponding peak in the XRD spectrum.

D = Kλ/β cosθ(3)

#### 3.2.13. Adsorption Isotherm Studies

The concentrations of the BSA solution were determined at known concentration intervals. A total of 0.1 g CS/β-CDP composite membrane was added to adsorb the BSA. The concentration of the BSA was from 0.2 mg/mL to 1.4 mg/mL. The mixture was shaken for 1 h at room temperature. To apprehend the adsorption mechanism, Langmuir, Freundlich, and Temkin models were applied in Table 10.

#### 3.2.14. Kinetic Studies

The concentrations of the BSA solution were determined by knowing the time intervals. A total of 0.1 g CS/β-CDP composite membrane was added to adsorb the BSA. The time of adsorption of the BSA was from 10 min to 150 min. To apprehend the adsorption mechanism, pseudo-first-order, pseudo-second-order, and intraparticle diffusion kinetic models were applied in Table 11.

#### 3.2.15. Thermodynamic of Adsorption

The concentrations of the BSA solution were determined at known temperature intervals. A total of 0.1 g CS/β-CDP composite membrane was added to adsorb the BSA. The temperature of adsorption BSA was from 15 °C to 55 °C. The mixture was shaken for 1 h at room temperature. To apprehend the adsorption mechanism, thermodynamic parameters, including enthalpy changes, entropy changes, and Gibbs free energy changes of the BSA adsorption on CS/β-CDP composite membrane, were applied to Table 12 for lnKc vs. 1/T and obtaining ΔH and ΔS.

## 4. Conclusions

In addition to its potential for protein adsorption, the CS/β-CDP composite membrane also shows promise in the field of laminate film applications. The optimized CS and β-CDP mass ratio, membrane preparation temperature, and glutaraldehyde addition of the composite membrane make it suitable for use as a laminate film with desirable properties. The tensile strength of the CS/β-CDP composite membrane, measured at 6.37 ± 0.31 MPa, indicates its ability to withstand mechanical stress and strain without breaking. The elongation at a break of 3.79 ± 0.56% suggests its flexibility and resistance to deformation. Young’s modulus of 7.56 ± 0.78 MPa reflects its stiffness and ability to maintain its shape under applied stress. Furthermore, the contact angle of 91.23 ± 0.89°, which is significantly higher than that of pure CS membrane, indicates that the CS/β-CDP composite membrane has improved hydrophobic properties. This could make it suitable for use as a barrier film to prevent the permeation of water or other liquids, depending on the intended application. The swelling degree of the CS/β-CDP composite membrane, measured at 35.48 ± 1.27%, is significantly lower than that of pure CS membrane. This suggests that the composite membrane has reduced water uptake and improved dimensional stability, making it suitable for use in applications where swelling or dimensional changes may be undesirable. The adsorption isotherms of the CS/β-CDP composite membrane indicate that the adsorption of BSA is primarily monolayer adsorption and physical adsorption, which is consistent with scanning electron microscopy (SEM) observations. The adsorption kinetics suggest that the BSA adsorption process on the composite membrane is controlled by a mixed mechanism of membrane and particle diffusion, in agreement with Fourier transform infrared spectroscopy (FT-IR) and X-ray diffraction (XRD) analyses. Thermodynamic analysis reveals that the adsorption of BSA by the CS/β-CDP composite membrane is an endothermic, spontaneous, and entropy-increasing process. This indicates that the composite membrane has favorable thermodynamic properties for protein adsorption. Overall, the CS/β-CDP composite membrane shows potential for various applications, including protein adsorption and laminate film applications. Its desirable properties, such as high tensile strength, flexibility, hydrophobicity, dimensional stability, and favorable thermodynamics, make it a promising adsorbent technology for protein removal, with potential environmental benefits, wide availability of raw materials, and low-cost production. Additionally, its suitability as a laminate film opens up possibilities for applications in areas such as packaging, coatings, and other barrier film applications. Further research and development in these areas could lead to the commercialization and practical implementation of CS/β-CDP composite membranes in various industrial applications.

## Figures and Tables

**Table 1:** Response Surface Design of Experiments.

Factor	Level		
	−1	0	1
The mass ratio (CS: β-CDP)	1:1.5	1:2	1:2.5
Temperature (℃)	30	40	50
Glutaraldehyde (mL)	1	1.5	2

**Table 2:** The experimental results of the response surface.

	CS: β-CDP the Mass Ratio (A)	Temperature (B) °C	Glutaraldehyde (C) mL	Adsorption Capacity mg/g
1	1	1	0	17.6264 ± 0.35
2	0	0	0	44.4734 ± 0.24
3	0	1	−1	20.3549 ± 0.16
4	−1	1	0	10.1752 ± 0.65
5	1	0	1	6.2239 ± 0.24
6	1	0	−1	20.8845 ± 0.75
7	0	0	0	43.5690 ± 0.34
8	−1	0	1	6.2889 ± 0.66
9	0	0	0	43.0253 ± 0.24
10	1	−1	0	13.0875 ± 0.15
11	0	0	0	47.3997 ± 0.72
12	0	−1	1	13.3404 ± 0.63
13	−1	0	−1	7.4853 ± 0.45
14	0	0	0	44.7543 ± 0.12
15	−1	−1	0	15.6978 ± 0.49
16	0	1	1	26.9946 ± 0.34
17	0	−1	−1	24.5480 ± 0.16

**Table 3:** Significance and variance test table.

Source	Sum of Squares	df	Mean Square	F-Value	*p*-Value Prob > F	
Model	3560.16	9	395.57	55.39	<0.0001	**
A	41.29	1	41.29	5.78	0.0472	*
B	8.98	1	8.98	1.26	0.2991	
C	52.15	1	52.15	7.30	0.0305	*
AB	25.31	1	25.31	3.54	0.1018	
AC	45.32	1	45.32	6.35	0.0399	*
BC	79.63	1	79.63	11.15	0.0124	*
A2	1820.45	1	1820.45	254.89	<0.0001	**
B2	396.53	1	396.53	55.52	0.0001	**
C2	782.27	1	782.27	109.53	<0.0001	**
Residual	50.00	7	7.14			
Lack of fit	38.58	3	12.86	4.51	0.0899	
Pure Error	11.41	4	2.85			
Cor Total	3610.15	16				

Note: ** indicates highly significant (*p* < 0.01); * indicates significant (0.01< *p* < 0.05).

**Table 4:** Results of mechanical property.

	Tensile Strength (MPa)	Elongation at Break (%)	Young’s Modulus (MPa)
Pure CS membrane	4.95 ± 0.22	2.68 ± 0.17	5.12 ± 0.67
CS/β-CDP Composite membrane	6.37 ± 0.31	3.79 ± 0.56	7.56 ± 0.78

**Table 5:** Results of contact angle and swelling degree.

Films	Water Contact Angle (°)	Glycerol Contact Angle (°)	Swelling Degree (%)
Pure CS membrane	66.43 ± 1.13	74.68 ± 1.04	71.06 ± 1.34
CS/β-CDP Composite membrane	91.23 ± 0.89	98.62 ± 2.42	35.48 ± 1.27

**Table 6:** Adsorption isotherm parameters.

	Langmuir	Freundlich	Temkin
Parameter model	y = 0.0050x + 0.0121	y = 0.6941x + 4.3376	y = 12.8721x + 49.0612
R^2^	0.9932	0.9453	0.9565
Parameter	a = 82.6446, b = 2.3725	n = 1.4407, K = 76.5237	b = 12.8721, A = 45.2137

**Table 7:** Kinetic model parameters.

	Pseudo-First-Order	Pseudo-Second-Order	Intraparticle Diffusion
Parameter Model	y = −0.0221x + 3.7533	y = 0.6477x + 0.0177	y = 3.6024x + 5.0022
R^2^	0.7806	0.9874	0.9301
Parameters	k = 0.0221	a = 0.6477, b = 0.0177	k = 3.6024, C = 5.0022

**Table 8:** Adsorption thermodynamic parameters.

Temperature (K)	ΔG (kJ/mol)	ΔH (kJ/mol)	ΔS (J/mol·K)
288	−5.4181	66.0601	251.7563
298	−8.9981		
308	−12.2254		
318	−12.9653		
328	−13.37105		

**Table 9:** Single-factor experimental design.

Influencing Factors	Level				
Mass ratio (CS:β-CDP) Temperature (°C) Glutaraldehyde (mL)	1:1	1:1.5	1:2	1:2.5	1:3
	20 °C	30 °C	40 °C	50 °C	60 °C
	0.5 mL	1 mL	1.5 mL	2 mL	2.5 mL

**Table 10:** Adsorption isotherm equation.

Isothermal Equation	Langmuir	Freundlich	Temkin
Original equation	$q=\frac{abC}{1+bC}$	$q=KC^{\frac{1}{n}}$	$q=b\ln\left(AC\right)$
Linear equation	$\frac{1}{q}=\frac{1}{abC}+\frac{1}{a}$	$\ln q=\ln K+\frac{1}{n}\ln C$	q=bln⁡A+bln⁡C

**Table 11:** Kinetic model equation.

Dynamical Equation	Pseudo-First-Order	Pseudo-Second-Order	Intraparticle Diffusion
Original Equation	$\ln\left(q_{e}-q\right)=\ln q_{e}-kt$	$\frac{1}{q}=\frac{a}{t}+b$	$q=kt^{\frac{1}{2}}+C$

**Table 12:** Thermodynamic equation.

Thermodynamic Parameters	ΔH	ΔG	lnKc
Original equation	ΔH=ΔG+TΔS	$\Delta G=-RT\ln K_{c}$	$\mathrm{lnKc}=\frac{\Delta S}{R}-\frac{\Delta H}{RT}$

## Sample Availability

Not available.
